# Root system architecture reorganization under decreasing soil phosphorus lowers root system conductance of *Zea mays*

**DOI:** 10.1093/aob/mcae198

**Published:** 2024-11-12

**Authors:** Felix Maximilian Bauer, Dirk Norbert Baker, Mona Giraud, Juan Carlos Baca Cabrera, Jan Vanderborght, Guillaume Lobet, Andrea Schnepf

**Affiliations:** Institute of Bio- and Geoscience: Agrosphere (IBG-3), Forschungszentrum Jülich GmbH, 52428 Jülich, Germany; School of Engineering and Natural Sciences, University of Iceland, Reykjavík, Iceland; Jülich Supercomputing Centre, Forschungszentrum Jülich GmbH, 52428 Jülich, Germany; Institute of Bio- and Geoscience: Agrosphere (IBG-3), Forschungszentrum Jülich GmbH, 52428 Jülich, Germany; Institute of Bio- and Geoscience: Agrosphere (IBG-3), Forschungszentrum Jülich GmbH, 52428 Jülich, Germany; Institute of Bio- and Geoscience: Agrosphere (IBG-3), Forschungszentrum Jülich GmbH, 52428 Jülich, Germany; Institute of Bio- and Geoscience: Agrosphere (IBG-3), Forschungszentrum Jülich GmbH, 52428 Jülich, Germany; Institute of Bio- and Geoscience: Agrosphere (IBG-3), Forschungszentrum Jülich GmbH, 52428 Jülich, Germany

**Keywords:** *Zea mays*, root system conductance, phosphorus, FSPM, root system architecture

## Abstract

**Background and Aims:**

The global supply of phosphorus (P) is decreasing. At the same time, climate change is reducing the availability of water in most regions of the world. Insights into how decreasing P availability influences plant architecture are crucial to understanding its influence on plant functional properties, such as the root system’s water uptake capacity.

**Methods:**

In this study, we investigated the structural and functional responses of *Zea mays* to varying P fertilization levels focusing especially on the root system’s conductance. A rhizotron experiment with soils ranging from severe P deficiency to sufficiency was conducted. We measured the architectural parameters of the whole plant and combined them with root hydraulic properties to simulate time-dependent root system conductance of growing plants under different P levels.

**Key Results:**

We observed changes in the root system architecture, characterized by decreasing crown root elongation and reduced axial root radii with declining P availability. Modelling revealed that only plants with optimal P availability sustained a high root system conductance, while all other P levels led to a significantly lower root system conductance, under both light and severe P deficiency.

**Conclusion:**

We postulate that P deficiency decreases root system conductance, which could mitigate drought conditions through a more conservative water use strategy, but ultimately reduces biomass and impairs root development and overall water uptake capacity. Our results also highlight that the organization of the root system, rather than its overall size, is critical for estimating important root functions.

## INTRODUCTION

The exploitation of finite natural resources poses new challenges to agriculture. The supply of phosphorus (P), a vital nutrient derived from finite resources, will decrease (Marschner, 2011). The predicted time for ‘peak phosphorus’, i.e. the time at which global P production reaches its maximum due to the depletion of reserves and declines again immediately afterwards, is estimated around the early to mid-21st century (Reijnders, 2014). Additionally, excessive use of P fertilizer significantly impacts the environment by contributing to eutrophication, which harms open water bodies and leads to aquatic plant and algae growth, impairing water quality for other organisms and limiting water use for drinking, recreation and industry (Randall, 2003). Especially in lakes, rivers, estuaries and coastal oceans, over-enrichment with P is a widespread problem (Carpenter *et al.*, 1998). Most of the P stored in water bodies originates from agricultural and urban activities. P fertilizers dissolve quickly, releasing P faster than plants can absorb it. P fertilizers, bound on loose soil particles, are highly prone to being lost by erosion. P that is not used by plants or is subject to runoff losses is immobilized in the soil and subsequently is no longer available for plants. For these reasons, a reduction of P fertilization is required (Hussain *et al.*, 2021).

Concurrently with the impending shortage of P, climate change is anticipated to lead to a scarcity of water across various regions around the globe (Gosling and Arnell, 2013). In view of this future water shortage, it is therefore crucial to gain an advanced understanding of how the reduced availability of soil P affects the plant’s architecture, specifically functional alterations related to the plant’s capacity for water uptake through its root system (Fry *et al.*, 2018).

*Zea mays* is one of the most important crops worldwide and crucial for human nutrition (Ranum *et al.*, 2014). Maize is sensitive to P deficiency and it is known that canopy development is inhibited by P deficiency, leading to yield decline. The plant’s architecture changes under soil P limitation. P deficiency is often associated with reduced growth and rigid appearance of shoots. Limited soil P availability also induces changes in root architecture. Studies report different morphological changes, such as the inhibition of primary root growth, shallower axial root angle, or various changes in lateral root growth, for example the reduction of lateral root growth in the field, but also an increase in lateral branching in plants with few axial roots (zero-order roots) (Borch *et al.*, 1999; Zhu and Lynch, 2004; Marschner, 2011), often resulting in a higher root to shoot biomass ratio (Lynch *et al.*, 2005). Furthermore, an increase in crown root number has been reported to be beneficial under P deficiency (Sun *et al.*, 2018). A reduced root radius was described as a response of *Z. mays* to reduced soil P availability in soil (Sheng *et al.*, 2012; Zhang *et al.*, 2012). However, no direct functional relationships between root system responses to P availability and root system functions have yet been established. Additionally, under field conditions, most plant responses are measured in rather coarse metrics and do not provide direct response functions (Lopez *et al.*, 2023). Although a variety of different plant responses were reported, it remains uncertain which parameters (non-aggregated, directly measurable attributes, such as type-dependent root length and number) have a direct impact on aggregated structural and functional root system traits, for example total root system volume or the plant’s water uptake capacity.

The root system architecture and anatomy are the main factors that are important for the plant’s water uptake capacity (Steudle, 2001). Root plasticity refers to the ability of plant roots to alter their growth depending on environmental conditions. Root architecture refers to the spatial and temporal distribution of roots within the soil. The root system can be described as an assembly of cylindrical root segments, and the root architecture defines the shapes of the individual segments, such as their length, radius and orientation, as well as how they are connected. Root anatomy relates to the internal structure of the root. Together, they govern the root hydraulic properties. The root hydraulic properties determine the plant’s capacity to channel water from the soil to the roots and then to the above-ground organs. Root system conductance (*K*_rs_) is a property of the root system and defines the absorptive capacity of the entire root system. As it is an intrinsic property of the root system, it does not depend directly on the surrounding soil environment. Consequently, *K*_rs_ is not conditioned by the characteristics of the perirhizal zone, the region surrounding the roots where radial symmetric flow and hydraulic gradient are generated by root water uptake (Vanderborght *et al.*, 2024). However, *K*_rs_ depends on the size and age of the root system. Since ageing root architecture development depends on environmental conditions, variations in *K*_rs_ depend indirectly on the soil environment (Meunier *et al.*, 2017; Baca Cabrera *et al.*, 2024). The variability in *K*_rs_ can be very high; for example, in young maize (up to 1 month), *K*_rs_ can vary from 7.00 × 10^−5^ to 2.37 × 10^−2^ cm^2^ d^−1^ (Baca Cabrera *et al.*, 2024). In order to relate *K*_rs_ to the size, age and architecture of the root system, it is necessary to know the hydraulic properties of the individual root segments that make up the whole root system. The radial hydraulic conductivity (*k*_r_) of a root segment is a measure of the root’s ability to take up water from the soil into its vascular system. *k*_r_ represents the water uptake potential by a root segment for a specific root surface and water potential gradient between the xylem and the outer surface of the root. The axial hydraulic conductance (*K*_x_) relates to the efficiency of water transport along the length of the root’s main axis (Steudle, 2001). It represents the ratio of the axial water flow in a segment (*J*_x_) to the potential gradient along the segment. Both *k*_r_ and *K*_x_ are intrinsic properties of the roots. *k*_r_ is often treated as an intensive property, i.e. it does not depend on the size or radius of the root segment. However, this is debatable as *k*_r_ can decrease with increasing root radius and cortical thickness, increasing the transport distance and, hence, the resistance to flow. *K*_x_ is an extensive property since *K*_x_ increases with an increasing cross-sectional area of the xylem tissue. *k*_r_ and *K*_x_ depend on the properties of the root tissues (cortex, xylem, casparian band), and changes in these properties at the cellular and organ levels can impact the root’s overall hydraulic function, which might alter the root system conductance. A change in *K*_rs_ affects the plant’s ability to uptake water (Meunier *et al.*, 2020). Many different environmental influences, such as drought or salinity, lower *K*_rs_ (Aroca *et al.*, 2011). P deficiency was also suggested to be an influencing factor for lowering *K*_rs_ in different species (Shanguan *et al.*, 2005; Mu *et al.*, 2006; Li *et al.*, 2009). We know that anatomical changes in the roots of *Z. mays* under P deficiency reduce the root hydraulic conductivity in very young plants, but it has also been shown that line-specific differences in the anatomical formation can strongly influence the *K*_rs_ changes caused by P deficiency (Fan *et al.*, 2007; Rishmawi *et al.*, 2023). In general, the relationship between soil P availability and key architectural root system parameters that drive changes in *K*_rs_ is not well understood. Moreover, *Z. mays* has rarely been the object of studies investigating the influences of P deficiency on *K*_rs_. Continuous data showing changes in conductance of the whole root system to soil P limitation over time are lacking but would be helpful to understand the influence of decreasing soil P on *K*_rs_. However, with experimental set-ups, it is especially challenging to quantify solely the effects of soil P limitation on whole crop and canopy development and its consequences on relevant physiological processes, such as water uptake-related functions. Especially for *k*_r_, experimental measurements require complex set-ups, such as a root pressure probe (Frensch and Steudle, 1989), measuring water flow of pruned roots within a pressure chamber (Zwieniecki *et al.*, 2002) or using a high-pressure flow meter device on whole root systems for root system conductance, as proposed by Tyree *et al.* (1994). The necessity of measuring *k*_r_ at several locations, in case of its variation along the root axis or for various root types, makes its experimental evaluation more challenging. Inverse modelling is a newer, additional method to obtain *k*_r_ and *K*_x_ values (Couvreur *et al.*, 2018).

Functional–structural plant models (FSPMs) are a suitable tool to help investigate and interpret the reaction of a plant to a changing environment, such as the absence of a crucial nutrient. They can bridge the gap between the (sub-)organ and whole plant level and thus simulate mechanistically emerging plant phenotypes caused by the interaction of processes at smaller scales, such as the effect of radial and axial water fluxes through root segments on whole plant water uptake. Indeed, FSPMs are computational frameworks that simulate plant growth by integrating physiological functions with (3D) structural representations of plant organs. In the context of P, FSPMs have already been used to test hypotheses regarding changes in the root architecture of *Z. mays*, such as a greater inter-lateral distance (Postma *et al.*, 2014) and a higher amount of seminal roots regarding its advantages for P uptake (Perkins and Lynch, 2021).

Although it has already been shown that root architecture and shoot size adaptation are affected by soil P availability, transferring these findings directly to the sub-organ level is very complex without a more detailed experimental investigation of how architecture changes at a high spatio-temporal resolution. Previous studies had, however, a coarse temporal or spatial resolution or focused on specific organs (Sun *et al.*, 2018). Moreover, it is also suggested that potential reactions to P deficiency can already occur in very early growth stages (Brunel-Muguet *et al.*, 2014), whereas experimental studies focused on older plants (Pereira *et al.*, 2020). To the best of our knowledge, there are currently no studies investigating the whole plant’s architectural response of *Z. mays* to different levels of P availability, including the influence of this response and its consequences for *K*_rs_.

This work aims to understand which root and shoot architectural parameters are responding to four decreasing soil P levels from sufficient to severely deficient and how this will affect the plant’s root system capacity for water uptake. Therefore, this study has two main objectives:

1. To identify experimentally which structural parameters of maize organs show the strongest responses to soil P availability.
2. To parameterize and use *Z. mays* FSPMs from experimental data to analyse how root system conductance in maize adapts to the different soil P availability levels.

## MATERIALS AND METHODS

### Experimental set-up

Five *Zea mays* cv. B73 plants per treatment were grown in rhizotrons (80 × 30 × 2 cm) (Pfeifer *et al*., 2014) under four levels of soil P availability, hereafter called P0, P1, P2 and P3. The experiment was conducted in a glasshouse at the Forschungszentrum Jülich GmbH, Germany (50°54ʹ36″N, 6°24ʹ49″E) from May to June 2022. As substrate, a P-deficient luvisol soil from the ‘Dikopshof’ long-time fertilization trial (Wesseling, Germany) was used (Schellberg and Hüging, 1997). The initial plant-available P concentration in soil [P extracted according to the calcium–acetate–lactate (CAL method)] was 1.8 mg P per 100 g soil (P0). The soil was fully enriched by all other nutrients and sufficiently supplied with demineralized water, so P was the only limiting factor for plant growth. The substrate was additionally fertilized (45 % P_2_O_5_, Triplesuperphosphate). The resulting soil P concentration was respectively 3.3 mg 100 g^−1^ for P1, 4.6 mg 100 g^−1^ for P2 and 7.7 mg 100 g^−1^ for P3. Together with P0, these four different soil P levels represent the different P content classifications for agricultural soils, low B to D range, as proposed by VDLUFA (Verband deutscher landwirtschaftlicher Untersuchungs- und Forschungsanstalten) (Wiesler *et al*., 2018). In the context of agricultural applications, P0 is in the range of severe, P1 of strong (B) and P2 of mild (low C) P deficiency, while P3 is P sufficient (D). Two seeds were planted in the rhizotrons and directly after germination of the first seed, the other seed was removed. The soil in the rhizotrons was saturated with demineralized water before the experiment began. In the first 2 weeks, 75 mL H_2_O d^−1^ and the following 2 weeks 125 mL H_2_O d^−1^ was added from the top, following established protocols for this set-up (Pfeifer *et al*., 2014), with an additional 50 % safety margin incorporated to ensure sufficient water supply for the specific soil and crop used in this study. Daily stress monitoring confirmed that plants did not experience water stress, as water availability was deliberately kept high to avoid confounding factors. To obtain a high temporal resolution, imaging was first performed daily and, after 3 weeks, every 2 d. The measurements were performed until 28 d after sowing (DAS).

To phenotype the roots, daily imaging of the root system was performed with a ‘PhotoBox’ equipped with a high-resolution camera (EOS 70D; 14 mm APS-C, Canon Inc., Tokyo, Japan), where the rhizotron was always located at the same position, avoiding distortion and image-shift (Pfeifer *et al.*, 2014). This allowed us to take high-resolution images of the whole growing root system. During the experiment, the rhizotrons are stored in boxes at 45° inclination so that the root system will grow towards and along the window of the rhizotron. The windows remained covered and heat-shielded between the measurements, so the roots grew in a dark and heat-isolated environment. To obtain information about the shoot architecture of the maize plant, we performed a high-resolution 2D-RGB measurement with a fixed position horizontally to the plant. The camera (X-S10, Fujifilm Holdings K.K, Tokyo, Japan) was equipped with a fixed focal length lens (35 mm APS-C, Fujifilm Holdings). To ensure good image processing, a uniform blue background was installed. During the measurement, the rhizotron was fixed at an angle of 45° to provide a vertical positioning of the maize shoot. To ensure detailed and accurate data collection, shoot imaging was conducted just prior to imaging of the roots. At the end of the experiment, a destructive biomass measurement was performed (Supplementary Data Fig. S1).

### Image processing

**Fig. 1.**
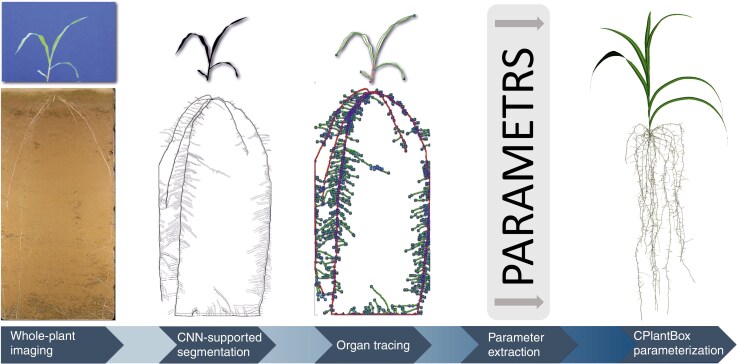
Workflow from experiment to CPlantBox model parameterization.

The data obtained from root and shoot are available as 2D RGB images (shoot: JPG, 2080 × 2080 px; root: JPG, 2268 × 4862 px). To facilitate analysis of the images, a mostly automated image-processing pipeline was established, streamlining the CPlantBox model parameterization from the experimental data (Fig. 1). The first step of image analysis was the segmentation of the targeted organ. The shoot image analysis pipeline started with the segmentation of the maize crop shoot. This was performed by a colour-threshold-filter algorithm written in Python based on the OpenCV wrapper PlantCV and the OpenCV library itself (Gehan *et al.*, 2017). The blue background was removed using a colour-based filter and only the predominantly green-to-red coloured plants were still present after filtering. Then, a semi-automated detection with ‘Root System Analyser’ (Leitner *et al.*, 2013) was performed. The parameters used for CPlantBox were directly derived from ‘Root System Analyser’. We used the procedure already successfully applied in Yu *et al.* (2024). For the root system part, we adapted the method from Bauer *et al.* (2022) to segment the roots in the image with a deep neural network model trained with ‘RootPainter’ (Smith *et al.*, 2022). We trained the neural network to ignore small gaps in the root system. However, since some gaps remained, we added a feature to ‘RootPainter’, allowing manual correction of segmentation errors and tracing the root by hand if needed. The ‘RootPainter’ add-on allowed us to analyse time series by transferring the segmentation of an image to the next consecutive image in the time series and only adding the additional segmented roots to the previous segmentation. The segmentation results were complete 2D binary root systems. The next processing step was the automated root tracing. ‘Root System Analyser’ directly provided the input parameter usable for CPlantBox, by manually choosing axial roots and automatically detecting the laterals. Finally, an RSML file (Root System Marker Language) for every root system and time step was produced by ‘Root System Analyser’ (Lobet *et al.*, 2015). The first root was always flagged as the primary root. To discriminate between crown roots and all other root types, the crown roots were manually flagged in the RSMLs with ‘Smart Root’ (Lobet *et al.*, 2011).

### CPlantBox parameter extraction

CPlantBox is a modelling platform that can simulate the morphology and 3D topology of the plant and, among other processes, plant and soil water fluxes (Giraud *et al.*, 2023). To use the CPlantBox modelling framework, plant parameters obtained from real plants are required to create a structure as either a virtual copy of an existing plant or a stochastic variation of a plant, representing the parameterized cultivar or line, respectively (Schnepf *et al.*, 2018; Zhou *et al.*, 2020). In terms of plant topology, it is possible to reduce the whole plant architecture to a handful of key parameters that are the input to calibrate CPlantBox. A precise parameterization of every organ type (e.g. leaf, basal roots) of the shoot and root system is required. This includes plant age at organ emergence, maximal length and initial elongation rate of stem, leaf and every root type. Depending on the organ, initial growth angle, radius, tropism, and branching distance and pattern have to be defined (Supplementary Data Fig. S2). These parameters were used as direct model input to simulate the plant structure. We furthermore have parameters that describe general root system traits, such as (first) initiation time, maximal count and appearance probability of different lateral root types, and seed position. We also have organ-specific parameters, which had to be measured and calculated for every organ sub-type. Regarding the shoot, this only applied to the leaf and stem. For the root system, specific parameter-ensembles were derived for every root type, respectively primary embryonic root (primary root), seminal roots, crown roots and lateral roots. For maize, there also exist two different types of lateral roots (Heymans *et al.*, 2021). We subdivided first-order laterals into l-(long) laterals, which have branching roots, and s-(short) laterals. In a CPlantBox simulation, each parameter is determined using the average value (mean) and variability (s.d.) from all the data points provided for parameterizing that specific organ. A comprehensive list detailing the parameters, their abbreviations and the units of measurement is given in Table 1.

**Table 1.** Overview of organ parameters (and their units) that are used to calibrate models with CPlantBox, as used for this study (day: d; integer: int), adapted from Schnepf *et al.* (2018), Zhou *et al.* (2020) and Giraud *et al.* (2023).

Root	Abbreviation	Unit	Shoot	Abbreviation	Unit
Planting depth	*depth*	cm	Nodal growth implementation	*nodalGrowth*	int [0,1]
First emergence of seminal roots	*first_B_*	d	Time period between leaves	*delayLat*	d
Time period between basal roots	*delay_B_*	d	Rotation of leaves around stem	*RotBeta*	int [0-1]
Max. no. of basal roots	*max_B_*	int	Shape type of leaves	*shapeType*	int [0,1]
First occurrence of crown roots	*first_SB_*	d	Petiole width	*Width_petiole*	cm
Time period between shoot-born roots per root crown	*delay_SB_*	int	Max. area of leaf	*areaMax*	cm²
No. of shoot-born roots per crown	*n_c_*	d	Geometry of the leaves	*leafGeometry*	*array*
Distance between crowns along the shoot	*dz_S_*	cm	Length of petiole	*l_b_*	cm
Root radius	*a*	cm	Stem radius	*a*	cm
Insertion angle	θ	rad	Insertion angle leaf	θ	rad
Length of basal zone	*l_b_*	cm	Length of stem until the first leaf	*l_b_*	cm
Apical delay	*l*_delay_	cm d^−1^	Length of leaf blade	*l_a_*	cm
Initial elongation rate	*r*	cm d^−1^	Init. elongation rate	*r*	cm d^−1^
Max. root length	*l*_max_	cm	Max. length	*l*_max_	cm
Tropism type^1^	*type*	int [0–3]	Tropism type^1^	*tropsimT*	int [0–5]
Tropism strength	*N*	int	Tropism strength	*tropsimN*	int
Root successor type	*successor*	type, %^2^	Successor	*successor*	type, %^2^
Type of root elongation^3^	*gf*	int [0,1]	Type of elongation^3^	*gf*	int [0,1]
Root lifetime	*rlt*	d	Lifetime	*rlt*	d
Max. segment length	*dx*	cm	Max. segment length	*dx*	cm

^1^Plagio-, gravi-, exo-, chemo-, hydro, antigravi- or age-dependent-tropism.

^2^Probability of emergence

^3^Negative exponential or linear growth.

The static root model parameters were directly derived from the RSMLs (Table 2). For the initial elongation rate parameter (*r*) a curve fitting was performed according to eqn (1) (Schnepf *et al.*, 2018). We assumed a maximal root length (*l*_max_) of 139 cm from the literature and fitted *r* only (Ordóñez *et al.*, 2018; Qiao *et al.*, 2019). General root system parameters, such as the amount and delay of seminal and crown roots, were evaluated manually from the rhizotrons. For leaves, we also considered negative exponential growth, according to eqn (1). Growth data from leaves that had not yet reached the phase of declining daily elongation rate were not used for the computation of *r*.

**Table 2.** Overview of initial root system architectural parameters for the distinguished soil P regimes. These parameters describe the initiation time, maximal count and appearance probability of the different lateral root types, the seed position and simulation time.

Root system parameter	P0	P1	P2	P3
*first*_*B*_ [d]	3.6	3.6	3.0	4.0
*delay*_*B*_ [d]	1.0	1.0	1.0	1.0
*max*_*B*_ [–]	3.5	3.5	3.5	3.5
*first*_*SB*_ [d]	8.6	9.4	9.2	8.2
*delay*_*SB*_ [d]	1.0	1.0	1.0	1.0
*delay*_*RC*_ [d]	7.4	6.6	6.3	6.2
*n*_*C*_ [–]	3.0	3.6	3.4	3.0
*seedPos* [*x*, *y*, *z*]	[0.0, 0.0, −3.0]			
*simulationTime* [d]	28			
*successor probability on axial roots*				
[l-lateral; s-lateral]	0.04; 0.96	0.05; 0.95	0.05; 0.95	0.05;0.95


$$\begin{array}{l} \begin{array}{l} l_{exp}\left(t\right)=l_{max}\left(1-e^{-\frac{r}{l_{max}}t}\right)\; \end{array} \end{array}$$ (1)

where *t* is time (d), *l*_max_ is the maximal length (cm) and *r* is the initial elongation rate (cm d^–1^).

### K_rs_ calculation

To calculate the root system conductance and assess the water uptake of the plant, information about *K*_r_ (d^–1^) and *K*_x_ (cm^3^ d^–1^) is required (Meunier *et al.*, 2018). The root hydraulic properties vary strongly among species but also among genotypes of the same species (Rishmawi *et al.*, 2023). However, most functional–structural simulations for maize rely on time dynamic hydraulic conductivity profile values from Doussan *et al.* (1998), a study conducted 25 years ago that only covers two root types, as highlighted in subsequent studies (Javaux *et al.*, 2008; Postma *et al.*, 2017; Meunier *et al.*, 2020). Besides measuring the radial flow and root anatomy, hydraulic anatomy simulators integrated into new modelling software tools can assist in a more precise estimation of these values (Couvreur *et al.*, 2018; Passot *et al.*, 2019; Heymans *et al.*, 2020). This provides new possibilities, such as the hydraulic atlas of *Z. mays* cv. B73 of Heymans *et al.* (2021). With these parameters, the hydraulic properties of the root system can be defined and *K*_rs_ (cm^2^ d^–1^) can be calculated according to Couvreur *et al.* (2012):


$$\begin{array}{l} \begin{array}{l} K_{rs}=\frac{T_{act}}{\psi_{sr,ef\;f}-\psi_{collar}}\; \end{array} \end{array}$$ (2)

where $\psi_{sr,ef\;f}\;(cm)$ is the *effective* soil–root interface water potential felt by the roots, $\psi_{collar}\;(cm)$ is the plant collar potential and *T*_act_ (cm^3^ d^–1^) is the actual plant transpiration rate and the net sum of the radial water flow rates ($J_{r}$, cm^3^ d^–1^) in the roots and root segments that make up the root system, respectively, since no changes in plant water storage are taken into account. $\psi_{sr,ef\;f}$ is obtained following the method of Couvreur *et al.* (2012):


$$\begin{array}{l} \begin{array}{l} \psi_{sr,ef\;f}=SUF^{T}.\psi_{sr}\; \end{array} \end{array}$$ (3)

where *SUF*  (−) is the vector containing the standard uptake fraction, which is the ratio between the water uptake of each root segment and the total water uptake of the root system, and $\psi_{sr}$ is the vector of soil water potentials at each root–soil interface. $J_{r}$ is defined as:


$$\begin{array}{l} J_{r}=K_{r}\left(\psi_{sr}-\psi_{xyl}\right) \end{array}$$ (4)

$$\begin{array}{l} K_{r}=2\pi \;\;\;a_{organ}\;\;\;dl\;\;\;k_{r} \end{array}$$ (5)

where $K_{r}$ is the radial conductance (cm^2^ d^–1^) of a root segment with an infinitesimal length d*l* (cm), $a_{organ}$ is the organ radius and $\psi_{xyl}$ is the xylem water potential (cm). As we assume steady-state water flow with no plant water storage variations, $J_{r}$ is equal to the changes in axial water flow ($J_{x}$, cm^3^ d^–1^) along l, so we obtain:


$$\begin{array}{l} J_{r}=\frac{\partial J_{x}}{\partial l}\partial l \end{array}$$ (6)

$$\begin{array}{l} J_{x}=K_{x}\frac{\partial \psi_{xyl}}{\partial l} \end{array}$$ (7)

$$\begin{array}{l} K_{x}=\frac{\pi {a_{xyl}}^{4}}{8\mu \;} \end{array}$$ (8)

where $a_{xyl}$ is the equivalent xylem radius and *µ* (cm d^–1^) is the dynamic water viscosity, assumed equal to that of pure water at 20 °C. Note that we express water potentials in terms of water heads, as is common in models that simulate water flow in soils. Equations (4)–(7) give us a system of equations that are solved analytically using the method of Meunier *et al.* (2020), implemented in CPlantBox according to Giraud *et al.* (2023). The solution yields both $J_{r}$ and $\psi_{xyl}$ for a specific set of *k*_r_ and *K*_x_.

We calculated the root hydraulic properties *k*_r_ and *K*_x_ from the values published in Heymans *et al.* (2021) for *Z. mays* cv. B73 (Supplementary Data Table S1). We assumed that *k*_r_ and *K*_x_ did not change between the different P treatments. We assume here that *k*_r_ and *K*_x_ do not depend on the root radius; however, the changes in radii between P treatments were considered when calculating radial conductance (eqn 5). Although it has been shown that the aerenchyma structure can change under P deficiency (Fan *et al.*, 2007), the inter-line-specific differences in *k*_r_ and *K*_x_ are much higher in *Z. mays* than the aerenchyma re-formation under P deficiency (Rishmawi *et al.*, 2023). Furthermore, the aerenchyma re-formation of *Z. mays* cv. B73, with a no-P treatment under lab conditions, is reported to be still very moderate (Fan *et al.*, 2007). Finally, the few root hydraulic property data available for maize under P deficiency are hard to use for our model since they only take a single root type (primary root) into account and are measured for very young plants grown in a nutrient solution. The data from Heymans *et al.* (2021) are given as distance depending on the root tip distance and for every root type. The conversion from distance-dependent to age-dependent conductivity was done using eqn (9). For a specific distance from the root base *l* (cm), the corresponding root segment age [$\begin{array}{l} age\left(l_{exp}\right)\; \end{array}$, d] was calculated:


$$\begin{array}{l} \begin{array}{l} age\left(l_{exp}\right)=-\frac{l_{max}}{r}ln\left(1-\frac{l}{l_{max}}\right)\; \end{array} \end{array}$$ (9)

where *r* is the initial elongation rate, obtained from the experiments and eqn (1), *l*_max_ is the maximal root length and *l* is the current measured root length (from experimental data). In contrast to Doussan *et al.* (1998), we distinguished between primary root, seminal roots, crown roots, l-lateral roots and s-lateral roots. For the parameterization of shoot organs we followed the simpler approach of Lobet *et al.* (2014), where it was assumed that the radial stem conductivity was 0 and the axial stem conductance (cm^3^ d^–1^) is also calculated according to the Hagen–Poiseuille law (eqn 8). We finally calculated Krs (eqn 2) using the simulated plant architecture and the root hydraulic anatomy based on Heymans *et al.* (2021).

### Statistical analysis

All statistical analyses, besides a principal component analysis (PCA), were performed with Python 3.9.13. For significance testing of the experimentally measured parameter between P treatments, we applied a Shapiro–Wilk Normality Test and Levene’s Test for Equality of Variances, followed by an ANOVA and Tukey post-hoc test with the ‘scikit’ package (scikit-learn 1.4.2) (Pedregosa *et al.*, 2011). The results of the statistical test are summarized in Supplementary Data Table S2. All parameters with significant differences (*P* < 0.05) were included in the PCA, namely axial root radii, leaf elongation and crown root elongation, and we further added *K*_rs_, dry matter, P to dry matter ratio and P measured in soil. We clustered for the different P treatments and included the five repetitions per treatment. For curve fitting of the initial elongation rate parameter (*r*) and maximal length (*l*_max_), the ‘scipy’ package was used (Virtanen *et al.*, 2020). PCA was performed with R 4.3.1 (R Core Team, 2021) and the ‘FactoMineR’ package (Lê *et al.*, 2008). For linear regression models of the identified response parameter, the ‘sklearn’ package was used (Pedregosa *et al.*, 2011). Plots were created with the ‘matplotlib’ package (Hunter, 2007).

## RESULTS

### Plant structural responses to soil P level

**Fig. 2.**
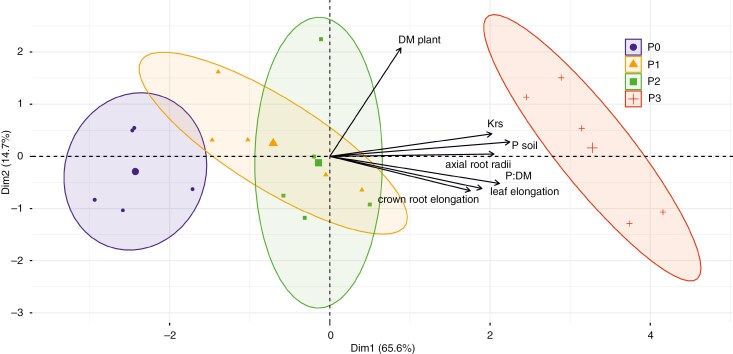
Principal component analysis (PCA) to identify the contribution of the plant parameters to the response of *Zea mays* to P deficiency. The large symbols correspond to the centroids for the different P treatments.

**Fig. 3.**
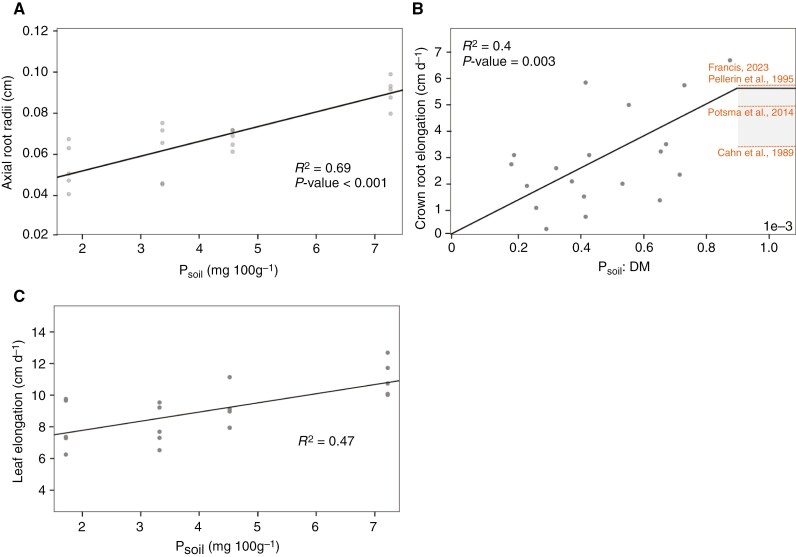
Response of axial root radii (A), crown root elongation (B) and leaf elongation rate (C) to different soil P availability levels.


The influences of P deficiency on the architecture of young root systems appear complex. Although we observed a reorganization in many different architectural root system traits, the clearest significant trends in root trait responses to P deficiency can be seen in the radius of axial roots and the elongation rate of crown roots (Supplementary Data Fig. S3A and 3B). The radii of axial roots increased significantly with the amount of P fertilized. Only for the initial leaf elongation rate did we find a significant architectural response of the shoot to soil P availability. The initial elongation rate was significantly higher for the highest P level (P3) compared to the two lowest P levels (P0 and P1) (Fig. 3C). Consequently, maximal leaf area showed an increasing trend as well. Although stem length and diameter also increased slightly with higher P supply, the differences between the soil P levels were not significant. The destructively measured root mass fraction (root biomass/plant biomass) showed a decreasing trend with increasing soil P availability (Fig. 3D). PCA clearly demarcated clusters for each P treatment level, with minimal overlap between the confidence ellipses. This suggests a strong grouping effect in our data, reflective of the distinct P treatments applied (Fig. 2). PCA further revealed that axial root radii are closely associated with soil P content, while crown root elongation showed a notable correlation with the soil P to plant dry matter ratio (*PB*, mg P hg soil^–1^ g biomass^–1^). A similar positive correlation was observed between soil P and leaf elongation rate. Therefore, we considered crown root elongation rate and axial root radii as plastic response parameters for root system changes. Additionally, the leaf elongation rate was considered as a shoot response to soil P availability.

For the radii of axial roots (*a*_ax_, cm) we found a direct linear relationship to P available in soil (Fig. 3A) within the measured upper boundary $\left(P_{max}\right)$ and lower boundary *P*_min_ (mg P hg soil^–1^) of soil P (mg P hg soil^–1^, described as in eqn 10):

$$\begin{array}{l} \begin{array}{lll} a_{ax}=\alpha_{a_{ax}}P+a_{P_{0}}, & \;\;\;\;\;P_{min}<P<P_{max}\; & \; \end{array} \end{array}$$ (10)

where parameter $\alpha_{a_{ax}}\left(cm\;hg\;soil\;mg\;P^{-1}\right)$ defines the increase in radii per unit of P in soil and $a_{P_{0}\;}\left(cm\right)$ the intercept of the response that represents the radii at the theoretical situation of no available P in soil. We found that the crown root elongation rate *r*_c_ (cm d^–1^) is a response to *PB* (eqn 11):


$$\begin{array}{l} \begin{array}{llll} PB & =\frac{P}{DM_{plant}},\; & \;r_{c} & =\left\{ \begin{array}{lll} \alpha_{r_{c}}PB, & 0<PB<PB_{max},\;\alpha_{r_{c}}PB_{max}, & PB\geq PB_{max}\; \end{array}\right.\; \end{array} \end{array}$$ (11)

where $\alpha_{r_{c}}\left(cm\;d^{-1}\;hg\;soil\;g\;\;\;biomass\;mg\;\;\;P^{-1}\right)$ is the increase in elongation per unit of *PB*. $PB_{max}\;\left(mg\;P\;\;\;hg\;soil^{-1}\;g\;biomass^{-1}\right)$ describes the maximal *PB* we measured, which, however, aligns with several maximal crown root elongation rates measured by other studies (Fig. 3B).

The initial leaf elongation rate ($r_{l}$, cm d^–1^) is a linear function of the P available in the soil and is described by eqn (12) (Fig. 3C):


$$\begin{array}{l} \begin{array}{lll} r_{l}=\alpha_{r_{l}}P+r_{P_{0}}, & \;\;\;\;\;P_{min}<P<P_{max}\; & \; \end{array} \end{array}$$ (12)

where $\alpha_{r_{l}}\;(cm\;hg\;soil\;d^{-1}\;mg\;P^{-1})$ is the increase in elongation per unit P, while $r_{P_{0}}\;(cm\;\;\;d^{-1})$ is the intercept at the theoretical situation of no soil P. $P_{min}$ and $P_{max}$ describe the lower and upper boundaries of P for the $r_{l}$ variations. Our observations revealed that leaf area was maintained for plants with higher P supply and sharply decreased at the two lowest soil P levels (Table 4). Root volume increased linearly with the amount of available soil P (Supplementary Data Fig. S4).

**Table 4.** Overview of shoot organ-specific architectural CPlantBox parameters, as described in Table 1, for the distinguished soil P regimes.

Parameter	P level type	P0		P1		P2		P3	
		Mean	s.d.	Mean	s.d.	Mean	s.d.	Mean	s.d.
*a*	Stem	0.187	0.01	0.16	0.02	0.166	0.014	0.13	0
*l*_*n*_	Stem	1.487	0.313	0.153	0.175	1.676	0.214	1.636	0.126
*r*	Stem	0.759	0.876	0.915	1.034	1	0.772	1.129	0.66
	Leaves	7.921	2.338	7.914	2.041	8.8	2.976	10.907	3.005
*l*_max_	Leaves	38.411	7.88	42.606	14.001	52.237	17.901	49.124	15.521
θ	Leaves	0.705	0.306	0.773	0.055	0.794	0.403	0.739	0.262
*delay*_*lat*_	Leaves	3		3		3		3	
*RotBeta*	Leaves	1		1		1		1	
*Width*_*Blade*_	Leaves	1.638		1.681		1.561		1.563	
*Area*_max_	Leaves	54.454		66.695		80.683		71.956	

**Fig. 4.**
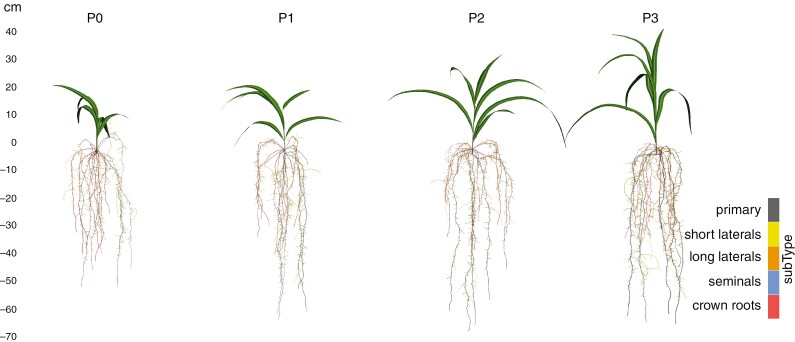
Simulated plant structure with CPlantBox for all soil P levels. The given subtypes correspond to the denomination of the root types.

For every soil P level, a complete CPlantBox parameter set was created for whole plant simulations (Fig. 4). A full list of the parameters, including root system-initializing parameters, as well as root- and shoot-specific parameters can be found in Tables 2, 3 and 4, respectively. We moreover created an FSPM, which simulates the dynamic growth of *Z. mays* cv. B73 under different soil P levels and modified only the identified key parameters (see section ‘P levels strongly influence axial root radius and crown root elongation’) according to the measured soil P levels. We compared the time-dependent simulated total root system volume and found no relevant absolute differences between the same treatments (Supplementary Data Fig. S4).

**Table 3.** Overview of root organ-specific architectural CPlantBox parameters for the distinguished soil P regimes and the parameter set (general) for simulation to evaluate the root system response parameter.

Parameter	P level type	P0		P1		P2		P3		General	
		Mean	s.d.	Mean	s.d.	Mean	s.d.	Mean	s.d.	Mean	s.d.
*a*	Primary	0.054	0.012	0.064	0.017	0.067	0.01	0.091	0.01	0.069	0.003
	Seminal	0.052	0.011	0.062	0.016	0.066	0.01	0.081	0.011	0.065	0.003
	Crown	0.061	0.016	0.059	0.02	0.066	0.014	0.066	0.003	0.063	0.007
	l-lateral	0.025	0.011	0.024	0.009	0.025	0.008	0.03	0.007	0.026	0.002
	s-lateral	0.025	0.007	0.028	0.01	0.025	0.008	0.04	0.012	0.030	0.002
*l*_b_	Primary	0.8	0.899	1.879	2.385	3.183	2.236	3.777	5.681	2.410	2.033
	Seminal	2.55	2.333	3.883	2.432	3.969	4.574	1.642	0.814	3.011	1.546
	Crown	3.161	2.454	7.216	7.564	3.473	2.487	3.924	3.025	4.444	2.468
	l-lateral	2.27	2.433	1.732	1.441	2.854	2.349	1.779	1.392	2.159	0.564
*l*_delay_	Primary	0.212	0.153	0.481	0.597	2.63	0.284	1.743	1.331	1.267	0.527
	Seminal	0.499	0.364	0.941	0.784	1.038	0.864	0.94	0.535	0.855	0.230
	Crown	0.194	0.124	0.666	0.625	0.547	0.476	0.666	0.428	0.518	0.210
	l-lateral	0.327	0.294	0.618	0.444	0.341	0.305	0.442	0.309	0.432	0.071
*r*	Primary	3.951	0.766	3.35	1.252	4.417	0.865	4.627	0.486	4.086	0.317
	Seminal	3.28	1.955	2.149	1.417	2.912	0.644	3.239	1.698	2.895	0.567
	Crown	2.981	2.693	2.556	2.902	2.29	2.146	4.886	2.583	3.178	0.319
	l-lateral	2.951	1.492	1.763	0.693	2.15	0.56	1.742	0.582	2.152	0.444
	s-lateral	2.555	2.479	5.078	4.814	5.292	4.982	5.97	5.168	4.724	1.263
*l*_max_	l-lateral	5.549	3.821	4.756	2.513	6.736	3.546	4.794	2.392	5.459	0.721
	s-lateral	1.631	1.596	1.341	1.15	0.84	0.452	1.238	1.123	1.263	0.472
θ	l-lateral	1.194	0.375	1.262	0.309	1.344	0.324	1.413	0.324	1.303	0.029
	s-lateral	1.194	0.375	1.37	0.346	1.396	0.327	1.413	0.324	1.343	0.023
*l*_*n*_	Primary	0.466	0.045	0.457	0.085	0.536	0.122	0.545	0.187	0.501	0.060
	Seminal	0.847	0.327	0.519	0.116	0.767	0.202	0.773	0.234	0.727	0.087
	Crown	0.847	0.327	0.628	0.244	0.811	0.419	0.754	0.124	0.760	0.125
	l-lateral	0.833	0.946	0.459	0.284	0.53	0.417	0.48	0.319	0.576	0.308

### Root system hydraulics

**Fig. 5.**
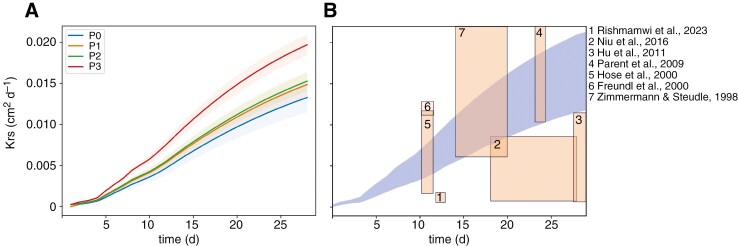
(A) *K*_rs_ calculated for each soil P level with 100 simulation runs (shaded areas show the standard deviation of the mean); (B) comparison of different studies investigating *K*_rs_ of *Zea mays* with our results (blue).

Based on the created FSPM, we calculated *K*_rs_. Our results indicate a close association of P in soil and *K*_rs_ (see Fig. 2 and Supplementary Data Fig. S5). The *K*_rs_ value for a root system under high to mild P deficiency was significantly lower than for the root system with a high P supply. After 28 d, the simulated mean *K*_rs_ (100 simulations) was between 0.014 and 0.016 cm^2^ d^−1^ for P0, P1 and P2, while P3 reached a mean *K*_rs_ of 0.021 cm^2^ d^−1^ at the same time point. The differentiation in *K*_rs_ between the treatments begins between 7 and 10 DAS (Fig. S5). Fig. 5 shows the temporal evolution of *K*_rs_ according to our simulations and in comparison with literature values.

## DISCUSSION

The study presented here focuses on two main points. First, we conducted a whole plant phenotyping experiment of *Z. mays* cv. B73 under various soil P availability conditions in rhizotrons to identify which architectural parameters of maize organs are responding most to variations in soil P availability. Second, we parameterized FSPMs based on the previously measured data to understand how root system conductance in maize adapts to the different soil P availability levels. With additional hydraulic property data (*k*_r_, *K*_x_) from Heymans *et al.* (2020), which was based on *Z. mays* cv. B73 anatomy, we were able to calculate *K*_rs_ according to the different structures of the root systems. This allowed us to explore the water uptake capacities of each root system under static soil conditions.

### P levels strongly influence axial root radius and crown root elongation

A detailed look at the architectural parameters of the plant revealed that initial leaf elongation reacted to P deficiency. We observed clear differences in maximal leaf area depending on the P content in the soil, which originated from significant differences in the initial elongation rate of the leaf between high and low soil P levels, indicating that the P deficiency was already an important limitation in the initial growing phase of early leaves. Finally, a reduction of the maximal leaf area might decrease maximal transpiration and photosynthesis. These results might not be surprising, as the P deficiency reaction of the plant is mainly linked to a rigid appearance of the shoot (Plénet *et al.*, 2000*a*, 2000*b*). However, the quantitative empirical values presented here and the derived response functions are valuable additions as, contrary to most studies, the functions are valid for soil P levels ranging from strongly limited to sufficient (Lopez *et al.*, 2023).

The influences of soil P limitation on the root system were more complex to disentangle, especially since there are maize genotypes that are considered P efficient and P inefficient. B73 is considered an inefficient line and is thus suitable for investigation on the reaction to P deficiency since possible reactions might be observed even under mild P stress (Kaeppler *et al.*, 2000). When responding to environmental conditions, several phenes interact, so phenotypic effects are not always clear to observe in a single organ, although they become more clear from a holistic perspective, when all organs are evaluated together (York *et al.*, 2013; Klein *et al.*, 2020). We call this effect a plastic reorganization of the root system. The reorganization effects are complex and our understanding of them is limited (Lynch, 2011). However, modelling approaches have already shown that an increasing amount of seminal roots might be beneficial for P uptake (Perkins and Lynch, 2021), although studies focusing on identification of quantitative trait loci of seminal root count and length report the opposite reaction of *Z. mays* cv. B73 under lab conditions (Zhu *et al.*, 2006). Our findings do not unequivocally support either of the divergent perspectives reported in the literature. However, the reduced radii of axial roots as a response to P deficiency aligns with previous observations. We could show that there is a high linear relationship between plant-available soil P and axial root radii (Fig. 3A) (Sheng *et al.*, 2012; Zhang *et al.*, 2012). Possibly the plant reduced carbon costs to invest it in other organs that might be beneficial for P uptake under soil P limitations or shifted biomass allocation to more metabolically efficient root classes (Lynch *et al.*, 2005; Lynch, 2019). Regarding crown root development, we know that a greater number of crown roots is beneficial under P deficiency (Sun *et al.*, 2018). However, past research has indicated that minimizing the amount of crown roots can substantially lower the metabolic expenses associated with root construction, allowing more metabolic energy to be allocated towards root extension (Gao and Lynch, 2016). Following the rhizoeconomic paradigm (Lynch *et al.*, 2005), this would suggest that an increased number of crown roots might already result in an initially reduced crown root elongation. Under conditions of nitrogen deficiency, it has already been observed that there is a decrease in the number of crown roots, which is accompanied by an increase in their elongation rate (Saengwilai *et al.*, 2014). For plants under P deficiency, the response of crown root elongation is less well-defined. We found that crown root elongation in young plants is already an important response parameter for *Z. mays* under P deficiency and has a negative linear response to decreasing soil P availability in soil. As P leaching to deeper soil strata is limited, lower crown root elongation under limiting soil P conditions would support enhanced topsoil foraging, which is considered to be beneficial for a greater P uptake (Lynch, 2019). However, we could not detect a significantly higher number of crown roots in plants under soil P limitation. This may be attributed to the limitations of the rhizotron set-up, which may impede the visibility of all crown roots. Alternatively, if crown root formation is indeed enhanced under P limitation, the effect may not be detectable until 28 DAS, necessitating a longer observation period for accurate quantification.

Overall, the observations in this study are not only meant to investigate shoot and root in terms of biological general validity but also to parameterize CPlantBox to obtain dynamic FSPMs under various soil P conditions and to obtain new findings from this model approach. To our knowledge, this is the first approach of a detailed whole-plant 3D FSPM parameterization of *Z. mays*.

### K_rs_ varies between fully fertilized and deficient plants, but not among those with severe to mild P deficiency

*K*_rs_ varies due to environmental conditions (Freundl *et al.*, 2000; Hose *et al.*, 2000; Baca Cabrera *et al.*, 2024). It is known that *K*_rs_ is influenced by drought (Parent *et al.*, 2009; Hu *et al.*, 2011) and osmotic stress (Niu *et al.*, 2016), but also due to genotypic differences (Rishmawi *et al.*, 2023). The *K*_rs_ values simulated with our *Z. mays* FSPM are in the same range as those given in these studies (7.00 × 10^−5^ and 2.37 × 10^−2^ cm^2^ d^−1^, see Fig. 5B). We found that when below a specific threshold, soil P limitations modulate the root system conductance, which might impact young plant vigour. Indeed, the *Z. mays* plants with the highest P supply had a significantly higher *K*_rs_ compared with the *K*_rs_ for the three lower soil P supply levels, indicating that, as soon as the plants suffer from P deficiency, the adjustment of the root architecture reduces their water uptake capacity. Interestingly, the degree of severity of the P deficiency has no significant influence on *K*_rs_. Changes in *K*_rs_ are not solely a consequence of architectural changes but rather the result of a combination of altered root architectural traits under P deficiency and the corresponding adjustments in root functional properties that govern water uptake capacity. In particular, soil P-related changes in root radii and crown root length, due to faster elongation (as shown by eqns 10 and 11), influence the root’s radial conductance, which significantly contributes to the observed changes in *K*_rs_. The reduction in axial root radius alone causes a reduction in the radial conductance (as shown by eqn 5). In addition, shorter roots have a non-linear reduction in *K*_rs_ because the relationship between root age, surface area, and *k*_r_ and *K*_x_ is non-linear (Doussan *et al.*, 1998; Meunier *et al.*, 2017). The non-linear response of *K*_rs_ to soil P in plants with slower growing crown roots may also be attributed to the higher axial conductivity found in the proximal parts of crown roots compared to other root types. This, combined with the fact that crown roots are connected to the shoot’s vascular system, enhances the propagation of xylem tension along crown roots, potentially providing benefits to faster growing crown roots than to slower growing ones (Ahmed *et al.*, 2018).

A biological implication could be that plants under soil P limitation with lower *K*_rs_ decrease transpiration later than plants with high *K*_rs_ since they have lower water use and soil water would not be depleted so quickly, which is beneficial to mitigate potential drought stress under certain conditions. In regions that can experience summer drought with only sporadic rainfall, maize plants with lower *K*_rs_ can continue transpiring at higher rates, which helps the plant to sustain higher carbon assimilation rates, potentially boosting yield if rains return to support the later growth stages. Already during the domestication process, *Z. mays* developed root systems with lower *K*_rs_ in regions with this climate pattern, such as southwest USA, suggesting a potential adaptive advantage to sporadic dry spells (Yu *et al.*, 2024). However, under sufficient water conditions, a high *K*_rs_ would be beneficial since the general capability of water uptake is higher. In rice, it has been demonstrated that at low soil P levels, the discrepancy in growth between well-watered and drought-stressed plants was insignificant compared to the difference observed in plants with sufficient soil P (De Bauw *et al.*, 2020).

These are new insights since studying *K*_rs_ experimentally at this high spatio-temporal scale is challenging due to the complex architecture of root systems, their dynamic interactions with varying soil environments, and the technical difficulties associated with accurately measuring water flow through roots under different conditions (Heymans *et al.*, 2020). With this approach, we have also shown that computational modelling is overcoming these challenges and could be a tool for improving our understanding of the dynamic modulation of root water uptake mechanisms under soil P starvation since all other investigation methods provide only static information at a certain time point and of specific parts of the root system (Shanguan *et al.*, 2005; Mu *et al.*, 2006; Li *et al.*, 2009; Yu *et al.*, 2024).

Our results showed changes in the root system architecture under soil P limitations. Root volume increases linearly with soil P. We identified decreasing axial root radii and crown root elongation as key parameters for root systems and leaf elongation as the main shoot response to soil P limitation. We combined these results into a functional–structural model to show that maximal potential water uptake capacity does not differ between plants with high and mild P deficiency but does differ between fully P-fertilized and P-deficient plants. Both root system anatomy and architecture are key to understanding root system function. Although root system architectural traits, such as volume, do increase linearly with soil P availability, the root system’s capacity to take up water does not follow the same trend. This underscores that root system organization is critical for its function rather than mere total size. The main reasons for this phenomenon are the non-linear relationship of *K*_rs_ with root surface area, root length and presumably volume (Meunier *et al.*, 2017; Baca Cabrera *et al.*, 2024), which is associated with the age dependence of *k*_r_ and *K*_x_ (Doussan *et al.*, 1998).

To guarantee better generalizability, it will be important to validate whether these results are applicable in field conditions and across different maize varieties. Experimentally testing water uptake with two or more contrasting soil P concentrations would provide additional validation. Measuring or simulating actual transpiration and root water uptake would be another way to validate the findings on the influence of soil P limitation on water uptake capacity. Further research is required to investigate the effects on older plants. Furthermore, an evaluation of how the local intrinsic root hydraulic properties themselves might change under P deficiency and information on the internal P concentration within different plant organs under various soil P conditions would be a valuable addition to the results presented here. This study does not fully account for the complexity and heterogeneity of all soil conditions and cases of extreme P over- or under-supply in natural settings, which can significantly affect nutrient availability and plant growth. While the focus on P is critical, it is important to consider interactions with other nutrients and how they collectively impact plant growth and development. The impact of varying environmental conditions beyond controlled settings on P stress responses is not fully explored and it would be beneficial if further future studies include a range of genetic diversity within *Z. mays* to understand how different genotypes respond to P deficiency.

## SUPPLEMENTARY DATA

Supplementary data are available at *Annals of Botany* online and consist of the following.

Fig. S1: Biomass (dry mass) [g] of shoot, root and whole plant depending on soil P level. Fig. S2: Schematic overview of the different organ parameters required for root and shoot calibration with CPlantBox. Fig. S3: (A) Axial root radii, (B) initial crown root elongation rate, (C) initial elongation rate of the leaves and (D) root fraction (root biomass:plant biomass) for different soil P availability levels. *P* < 0.05. Fig. S4: Total volume [$cm^{3}$] of simulated root systems with all parameters as measured and with all parameters set as the mean and only identified response parameters elongation of crown roots and axial root radii, set as a function of P level in soil. Fig. S5: *K*_rs_ for 7, 14, 21 and 28 DAS depending on soil P concentration, depending on the mean of 100 simulations. The error-bars display the standard deviation.

mcae198_suppl_Supplementary_Figure_S1

mcae198_suppl_Supplementary_Figure_S2

mcae198_suppl_Supplementary_Figure_S3

mcae198_suppl_Supplementary_Figure_S4

mcae198_suppl_Supplementary_Figure_S5

mcae198_suppl_Supplementary_Materials

mcae198_suppl_Supplementary_Tables_S1-S2

## Data Availability

All analysed data, code and model input files used for simulations and to plot the figures are publicly available and released in a GitHub repository https://github.com/Plant-Root-Soil-Interactions-Modelling/CPlantBox/releases/tag/Publication2024 in the folder /experimental/pdef. The image data are available at: doi.org/10.5281/zenodo.11384890. We further transferred the simulation set-up to a docker container for easy access (Supplementary Data).
