# Supplementary material for: Root system architecture reorganization under decreasing soil phosphorus lowers root system conductance of Zea mays
Source: Ann Bot. 2024 Nov 12;136(5-6):973–86. doi: 10.1093/aob/mcae198 (PMC12682837; doi:10.1093/aob/mcae198)
Supplement: mcae198_suppl_Supplementary_Materials [file mcae198_suppl_supplementary_materials.docx]

Supplementary Instruction

**Instruction to run Docker Container**

1. Install & start Docker (Docker Desktop is recommended)
2. To pull image: docker pull satraox/cplantbox-bauer.
3. To run the container: docker run -it cplantbox-bauer:latest
4. Go to data and simulation directory by: cd experimental/pdef
5. For simulation go to simulation directory: cd simulation
6. Run sim.py by: python3 sim.py
7. Results will appear in the same directory
8. For data visit the folder: response_parameter
