# Supplementary material for: Root system architecture reorganization under decreasing soil phosphorus lowers root system conductance of Zea mays
Source: Ann Bot. 2024 Nov 12;136(5-6):973–86. doi: 10.1093/aob/mcae198 (PMC12682837; doi:10.1093/aob/mcae198)
Supplement: mcae198_suppl_Supplementary_Tables_S1-S2 [file mcae198_suppl_supplementary_tables_s1-s2.docx]

Supplementary Tables

Supplementary Table 1: Overview of the hydraulic properties (*k*_r_ and *K*_x_) used to compute dynamic root system conductance (*K*_rs_), based on the simulated root system architecture.

|  | Primary |  | Seminal roots | | | Crown roots | | | Long laterals | | | Short laterals | | |
| --- | --- | --- | --- | --- | --- | --- | --- | --- | --- | --- | --- | --- | --- | --- |
| age | kr | Kx | age | kr | Kx | age | kr | Kx | age | kr | Kx | age | kr | Kx |
| 0.11 | 1.06E-04 | 0.0014 | 0.13 | 1.13E-04 | 0.001 | 0.11 | 1.34E-04 | 0.0061 | 0.24 | 1.18E-04 | 4.48E-05 | 0.3 | 1.29E-04 | 1.49E-05 |
| 0.16 | 1.06E-04 | 0.0014 | 0.19 | 1.13E-04 | 0.0011 | 0.16 | 1.34E-04 | 0.0061 | 0.38 | 1.18E-04 | 4.63E-05 | 0.53 | 1.30E-04 | 1.60E-05 |
| 0.22 | 1.06E-04 | 0.0015 | 0.26 | 1.13E-04 | 0.0011 | 0.22 | 1.34E-04 | 0.0061 | 0.51 | 1.18E-04 | 3.62E-05 | 0.92 | 1.29E-04 | 1.58E-05 |
| 0.27 | 1.06E-04 | 0.0015 | 0.32 | 1.13E-04 | 0.0011 | 0.27 | 1.34E-04 | 0.006 | 0.66 | 1.18E-04 | 4.87E-05 | >2.83 | 1.30E-04 | 1.58E-05 |
| 0.32 | 1.06E-04 | 0.0014 | 0.39 | 1.13E-04 | 0.0011 | 0.32 | 1.35E-04 | 0.0063 | 0.82 | 1.18E-04 | 3.97E-05 |  |  |  |
| 0.38 | 1.06E-04 | 0.0015 | 0.45 | 1.13E-04 | 0.0011 | 0.38 | 1.34E-04 | 0.0059 | 0.98 | 1.18E-04 | 4.29E-05 |  |  |  |
| 0.43 | 1.06E-04 | 0.0014 | 0.52 | 1.14E-04 | 0.001 | 0.43 | 1.35E-04 | 0.006 | 1.16 | 1.18E-04 | 4.66E-05 |  |  |  |
| 0.65 | 1.06E-04 | 0.0015 | 0.78 | 1.22E-04 | 0.001 | 0.65 | 1.35E-04 | 0.0058 | 2.02 | 1.18E-04 | 4.22E-05 |  |  |  |
| 0.87 | 1.06E-04 | 0.0014 | 1.05 | 1.22E-04 | 0.001 | 0.87 | 1.41E-04 | 0.0058 | 3.35 | 1.18E-04 | 3.86E-05 |  |  |  |
| 1.09 | 1.06E-04 | 0.0015 | 1.31 | 1.23E-04 | 0.001 | 1.09 | 1.41E-04 | 0.0067 | >6.28 | 3.75E-05 | 4.21E-05 |  |  |  |
| 1.54 | 1.14E-04 | 0.0013 | 1.58 | 1.23E-04 | 0.0009 | 1.31 | 1.42E-04 | 0.0074 |  |  |  |  |  |  |
| 1.77 | 1.14E-04 | 0.0014 | 1.85 | 1.23E-04 | 0.0009 | 1.54 | 1.43E-04 | 0.007 |  |  |  |  |  |  |
| 2 | 4.00E-05 | 0.0013 | 2.12 | 1.23E-04 | 0.0008 | 1.77 | 1.47E-04 | 0.0064 |  |  |  |  |  |  |
| 2.23 | 3.99E-05 | 0.002 | 2.4 | 1.24E-04 | 0.0008 | 1.99 | 1.48E-04 | 0.0068 |  |  |  |  |  |  |
| 2.69 | 3.99E-05 | 0.1614 | 2.67 | 1.23E-04 | 0.0009 | 2.22 | 1.48E-04 | 0.0069 |  |  |  |  |  |  |
| 2.93 | 4.00E-05 | 0.1633 | 2.95 | 4.22E-05 | 0.0008 | 2.69 | 1.44E-04 | 1.4277 |  |  |  |  |  |  |
| 3.17 | 3.68E-05 | 0.1718 | 3.23 | 4.23E-05 | 0.0008 | 2.92 | 5.28E-05 | 1.5169 |  |  |  |  |  |  |
| 3.41 | 3.68E-05 | 0.1736 | 3.52 | 4.24E-05 | 0.0009 | 3.16 | 5.28E-05 | 1.6072 |  |  |  |  |  |  |
| 4.63 | 3.89E-05 | 0.1618 | 3.8 | 4.25E-05 | 0.0009 | 3.4 | 5.31E-05 | 1.6917 |  |  |  |  |  |  |
| 7.25 | 4.10E-05 | 0.2438 | 4.09 | 4.26E-05 | 0.0009 | 4.63 | 5.08E-05 | 2.3449 |  |  |  |  |  |  |
| >10.12 | 4.29E-05 | 0.3336 | 5.57 | 3.93E-05 | 0.1122 | 7.24 | 5.24E-05 | 4.0946 |  |  |  |  |  |  |
|  |  |  | 8.71 | 4.16E-05 | 0.1015 | >10.11 | 5.67E-05 | 6.6086 |  |  |  |  |  |  |
|  |  |  | >12.16 | 4.31E-05 | 0.1505 |  |  |  |  |  |  |  |  |  |

Supplementary Table 2: Overview of statistical results for axial root radii, crown root elongation and leaf elongation for the P levels P0-P3. A Shapiro-Wilk Normality Test and Levene's Test for Equality of Variances were performed, followed by an ANOVA (n = 5 - 12).

|  |  | axial root radii | | | | crown root elongation | | | | leaf elongation | | | |
| --- | --- | --- | --- | --- | --- | --- | --- | --- | --- | --- | --- | --- | --- |
|  |  | P0 | P1 | P2 | P3 | P0 | P1 | P2 | P3 | P0 | P1 | P2 | P3 |
| Shapiro-Wilk Normality Test | F | 0.89 | 0.68 | 0.6 | 0.48 | 0.93 | 0.98 | 0.83 | 0.98 | 0.89 | 0.9 | 0.95 | 0.92 |
|  | *p* | > 0.05 | > 0.05 | > 0.05 | > 0.05 | > 0.05 | > 0.05 | > 0.05 | > 0.05 | > 0.05 | > 0.05 | > 0.05 | > 0.05 |
| Levene's Test for Equality of Variances | F | 1.09 | | | | 2.45 | | | | 1.07 | | | |
|  | *p* | > 0.05 | | | | > 0.05 | | | | > 0.05 | | | |
| ANOVA | F | 10.21 | | | | 4.73 | | | | 4.04 | | | |
|  | *p* | < 0.001 | | | | 0.01 | | | | 0.01 | | | |
